# *TFRC* promotes the proliferation, migration, and invasion of osteosarcoma cells by increasing the intracellular iron content and *RRM2* expression

**DOI:** 10.3389/fonc.2025.1567216

**Published:** 2025-05-29

**Authors:** Guoqing Ren, Jian Zhou, Yong Su, Qiang Yang, Jianmin Li

**Affiliations:** ^1^ Department of Orthopedics, Qilu Hospital of Shandong University, Jinan, Shandong, China; ^2^ Department of Orthopedics, Qingdao Hospital, University of Health and Rehabilitation Sciences (Qingdao Municipal Hospital), Qingdao, China; ^3^ Qingdao Hospital, University of Health and Rehabilitation Sciences (Qingdao Municipal Hospital), Qingdao, China

**Keywords:** TFRC, osteosarcoma, iron, RRM2, proliferation, migration, invasion

## Abstract

**Background:**

Multiple studies have shown that the transferrin receptor (*TFRC*) is highly expressed in various tumors, and it has been recognized as a cancer biomarker. However, its role in osteosarcoma(OS) has rarely been studied. The purpose of this study was to explore the role and mechanism of *TFRC* in the proliferation, invasion, and migration of osteosarcoma cells.

**Methods:**

First, we analyzed the expression of *TFRC* in OS and normal cells with an open database and evaluated the correlation between *TFRC* expression and overall survival in OS patients. Quantitative real-time PCR (qRT–PCR), Western blotting, and immunohistochemical staining were used to determine the expression level of *TFRC* in OS cell lines and tissues. *TFRC* was knocked down by lentivirus-mediated short hairpin RNA (shRNA) in 143B and U2OS cells. The effects of *TFRC* knockdown on OS cell proliferation, migration, and invasion, as well as its mechanism related to ribonucleotide reductase M2 (*RRM2*), were explored through a series of experiments. Nude mice were inoculated with xenogeneic OS cells to study the influence of *TFRC* knockdown on tumor growth *in vivo*.

**Results:**

*TFRC* was highly expressed in osteosarcoma, and its high level of expression was associated with poor overall survival in osteosarcoma patients. After *TFRC* was knocked down, the proliferation, migration and invasion ability of OS cells were significantly reduced, and *TFRC* knockdown effectively inhibited the growth of OS cells in xenograft experiments with nude mice. The knockdown of *TFRC* led to a decrease in the total intracellular iron content and a significant decrease in the protein expression of *RRM2*. The decrease in the proliferation, migration and invasion of osteosarcoma cells caused by *TFRC* knockdown was reversed by the addition of FAC or plasmids to overexpress *RRM2*.

**Conclusion:**

OS cells regulate proliferation, migration, and invasion by overexpressing *TFRC*, which increases the transport of iron into cells and increases the expression and activity of *RRM2*.

## Introduction

Osteosarcoma (OS) is a highly aggressive mesenchymal malignant bone tumor that affects mainly children and adolescents, with a second peak in people aged 60–80 years (1–3), and the incidence during adolescence is approximately 4.4 cases per million people per year (4). Given that current standardized neoadjuvant chemotherapy, extensive surgical resection, and adjuvant chemotherapy are widely used, the quality of life of OS patients has improved, with the 5-year survival rate increasing to 70% (5, 6). However, there has been no substantial breakthrough in treatment since the 1980s (1), and over the past four decades, there has been limited progress in terms of the OS survival rate due to early metastasis and chemotherapy resistance (6, 7). The 5-year overall survival rate of patients with metastatic OS is only approximately 20–40% (8), whereas for patients with recurrence, the prognosis is poor, with a survival rate of less than 20% (1). Therefore, further exploration of the new mechanisms involved in the occurrence and development of osteosarcoma and the search for new therapeutic targets have become topics of great interest in current osteosarcoma research.

Iron is an essential element for cellular activities, especially for rapidly proliferating tumor cells where the demand for iron significantly increases, also known as “iron addiction” (9, 10). Iron is involved in a variety of biological processes, including cellular respiration, energy metabolism, DNA synthesis, and redox reactions (11–13), and the dysregulation of iron metabolism often leads to the occurrence and development of tumors (9, 10, 14, 15). To meet the high demand for iron, tumor cells reshape iron metabolism pathways, resulting in the dysregulation of key proteins involved in iron metabolism and the overexpression of some iron uptake-related genes (16, 17).

Transferrin receptor 1 (TFR1) is an important transmembrane glycoprotein encoded by the *TFRC* gene that mainly regulates iron absorption by binding to transferrin (Tf), which is a key molecule in iron metabolism (13, 18–20). The expression level of *TFRC* is closely related to the iron demand of cells. Studies have shown that *TFRC* is highly expressed in a variety of tumors, including breast cancer, glioma, ovarian cancer, lung cancer, hepatocellular carcinoma, and colon cancer, and is considered a universal cancer marker (15, 21–25). This high expression of *TFRC* is closely related to the malignancy, invasiveness, metastasis and prognosis of tumors (13, 16, 26). However, its role in osteosarcoma has not been fully demonstrated.

Ribonucleotide reductase M2 (*RRM2*) is a subunit that regulates the activity of the enzyme ribonucleotide reductase (RNR), which is involved in the synthesis of DNA and is the rate-limiting step in the process of DNA synthesis (27, 28). *RRM2* is known to be overexpressed in ovarian, bladder and colorectal cancer (29–31). Elevated *RRM2* expression is a feature of many cancers, and a series of RNR inhibitors with different mechanisms can serve as effective drugs for cancer treatment (27). Iron is an essential metal cofactor for *RRM2* to form tyrosine radicals on Tyr122, which play a key role in the reductase activity of RNR (32). Therefore, we speculated that *TFRC* may play an important role in regulating the biological functions of tumor cells by modulating iron uptake and affecting the activity of *RRM2*.

We conducted experiments on the expression of *TFRC* in osteosarcoma, its influences on proliferation, migration, and invasion, and its mechanisms *in vitro* and *in vivo* to provide new ideas for the treatment of osteosarcoma. Our results revealed that *TFRC* is clearly overexpressed in osteosarcoma cells and tissues and that the knockdown of *TFRC* inhibits the transport of iron into osteosarcoma cells, reduces the total intracellular iron content, and inhibits the expression and activity of *RRM2*, thus inhibiting the proliferation, migration and invasion of osteosarcoma cells. Therefore, *TFRC* is a very promising target for osteosarcoma therapy.

## Materials and methods

### Bioinformatics database selection and analysis

We obtained bioinformatics data from multiple public databases. The GSE42352 dataset from the GEO platform (https://www.ncbi.nlm.nih.gov/geo) contains RNA sequences of 19 osteosarcoma cell lines and 12 mesenchymal stem cell samples (33, 34). We also used the GEPIA (http://gepia2.cancer-pku.cn/#survival) online platform to analyze the impact of *TFRC* and *RRM2* on overall survival in the sarcoma (SARC) dataset (35). The TARGET database (https://portal.gdc.cancer.gov/projects/TARGET-OS) provided the osteosarcoma (TARGET-OS) dataset, which contains comprehensive genomic features and survival information of 88 clinically annotated patients. The GTEx project (https://www.gtexportal.org/home/) provided 396 cases of genome expression and survival information of normal human bone and muscle tissue (36) for comparison with TARGET-OS. R software (v4.4) and the Limma software package were used to process the acquired data and perform differential expression analysis and survival analysis of target genes.

### Cell lines and cell culture

The human osteosarcoma cell lines (MNNG/HOS, U2OS, MG-63, and 143B) were gifted by Dr. Zhang from the laboratory of Jinan Central Hospital. The human osteoblast cell line hFOB1.19 was obtained from Procell Life Science & Technology Co., Ltd. The human osteosarcoma cell lines MNNG/HOS, MG-63, and 143B were cultured in Dulbecco’s modified Eagle medium (Gibco, Biochemical Products (Beijing) Co., Ltd.) supplemented with 10% fetal bovine serum (PAN, made in Germany) and 1% penicillin–streptomycin (Cytiva, Austria) at 37°C in a humidified incubator with 5% CO_2_. The human osteosarcoma cell line U2OS was maintained in complete McCoy’s 5A medium containing 10% fetal bovine serum (PAN, made in Germany) and 1% penicillin–streptomycin (Cytiva, Austria) at 37°C with 5% CO_2_. The human cell line hFOB1.19 was cultured in specialized culture medium for hFOB1.19 from Procell Life Science & Technology Co., Ltd., at 34°C with 5% CO_2_. The medium contained DMEM/F12 (PM150312), 0.3 mg/mL G418 (PB180125), 10% FBS (164210-50) and 1% P/S (PB180120).

### Establishment of stable TFRC-knockdown cell lines, plasmid construction and cell transfection

*TFRC*-shRNA and *TFRC*-shCtrl were synthesized and packaged into lentiviruses by OBiO Technology Corp., Ltd. (Shanghai, China). 143B and U2OS cells were transfected with synthetic lentivirus and 5 µg/mL polybrene (OBiO Technology, Shanghai, China) according to the manufacturer’s instructions, and the cells were observed under a fluorescence microscope (Nikon Ti2-U, Japan) and photographed 72 hours after transfection. The stably transfected cells were subsequently screened with 3 µg/ml puromycin (Sparkjade, Shandong, China), and related experiments were subsequently performed. The sequence of *TFRC*-shRNA was 5’-GCTGGTCAGTTCGTGATTAAA-3’, and the sequence of *TFRC*-shCtrl was 5’-CCTAAGGTTAAGTCGCCCTCG-3’. The cells were transfected with the *RRM2* overexpression plasmid and control plasmid (Nanjing Zebrafish Biotechnology Co., Ltd.) via KeygenMax 2000 Transfection Reagent (KeyGEN BioTECH, Nanjing, China) according to the instructions, and the medium was replaced with fresh medium 6 hours later. After 48 hours, the cells were observed under a fluorescence microscope, photographed, and harvested for assessment and subsequent related experiments.

### RNA isolation and quantitative real time PCR

Total RNA was isolated and purified from cells/tissues via the FastPure Cell/Tissue Total RNA Isolation Kit V2 (Vazyme, Nanjing, China) according to the manufacturer’s instructions. The RNA was reverse-transcribed into cDNA via HiScript III RT SuperMix for qPCR (Vazyme, Nanjing, China). The qRT–PCR was subsequently performed via ChamQ Universal SYBR qPCR Master Mix (Vazyme, Nanjing, China), and an ABI QuantStudio 3 Real-time PCR Detection System (Life Technologies) was used to obtain the data. Human GAPDH was chosen as an internal control for normalization to analyze the expression of target genes (37). All primers for these genes were synthesized by Sangon Biotech Co., Ltd. (Shanghai, China). The primers used were as follows: GAPDH-F[5’-GATTCCACCCATGGCAAATTC-3’], GAPDH-R[5’-CTGGAAGATGGTGATGGGATT-3’], TFRC-F[5’-TGAGGGAGGAGCCAGGAGAGG-3’], TFRC-R[5’-CTTGATGGTGCCGGTGAAGTCTG-3’]. The relative fold change in expression with respect to a reference sample was calculated via the 2**^-ΔΔCt^** method.

### Western blotting analysis

The cells and comminuted tissues were lysed in ice-cold RIPA buffer containing 1 mM PMSF (Sparkjade, Shandong, China) and phosphatase inhibitor (Solarbio, Beijing, China) for 30 minutes, and the lysates were subsequently centrifuged at 12,000 rpm for 10 minutes at 4°C. The liquid supernatant was collected, and protein concentrations were quantified via an Enhanced BCA Protein Assay Kit (Beyotime, Shanghai, China) according to the manufacturer’s instructions. The protein samples were mixed with 5× loading buffer and heated at 100°C for 10 minutes. Equal amounts of protein samples were subjected to 7.5% SDS–PAGE (Sparkjade, Shandong, China), separated, and transferred onto 0.2 μm PVDF membranes (PALL, USA) via standard procedures. The membranes were blocked in 5% nonfat milk for 1 hour at room temperature, incubated overnight at 4°C with specific primary antibodies diluted (anti-*TFRC*, 1:1000; anti-*RRM2*, 1:1500) with SuperKine™ Enhanced Antibody Dilution Buffer (Abbkine, Wuhan, China), and then incubated with goat anti-rabbit IgG (H+L) HRP or goat anti-mouse IgG (H+L) HRP (Sparkjade, Shandong, China) for 1 hour at room temperature. Protein bands were detected via a SuperFemto ECL Chemiluminescence Kit (Vazyme, Nanjing, China), and band intensities were quantified via ImageJ software. The primary antibodies against *TFRC* and *RRM2* were purchased from Abcam (Shanghai, China), and the primary antibodies against β-actin and GAPDH were purchased from Proteintech (Wuhan, China).

### Human tissue specimens and immunohistochemical staining analysis

Paraffin sections of human osteosarcoma and osteoblastoma samples were obtained from 30 osteosarcoma patients and 12 osteoblastoma patients admitted to Qilu Hospital of Shandong University from 2013 to –2022 and were used for immunohistochemical staining experiments. Written informed consent was obtained from all participants or their guardians. All study protocols were approved by the Ethics Committee on Scientific Research of Shandong University Qilu Hospital (permit number KYLL2023-06-090) and complied with the principles expressed in the Declaration of Helsinki. For immunohistochemical staining, paraffin sections were incubated overnight at 4°C with an anti-*TFRC* antibody (AB214039, Abcam; 1:500 for IHC staining analysis) or anti-*RRM2* antibody (AB172476, Abcam; 1:1000 for IHC staining analysis) after deparaffinization, rehydration, antigen retrieval, and endogenous peroxidase blockade. The paraffin sections were incubated with the secondary antibody (ZSBG-BIO, PV6000) at room temperature for 30 minutes and then developed with a DAB kit. The immunoreactivity of each tissue slice was assessed by two separate experienced pathologists. The IHC staining score was determined according to the staining intensity and degree of the Fromowitz criterion (38).

### Cell proliferation assay

143B or U2OS cells (4 × 10^4^/ml) were seeded into 96-well plates. Then, 90 μl of fresh culture medium without FBS and 10 μl of Cell Counting Kit-8 (CCK-8) solution (Dojindo, Japan) were added to each well at 37°C for 1.5 hours. The absorbance values were measured at 450 nm via a microplate reader (SpectraMax Plus384, Molecular Devices, USA). For the ferric ammonium citrate (FAC)-related assays, complete media with or without 100 µM FAC was replaced for each well 6 hours after these cells were seeded, and the remaining experimental steps were the same as before. All the experiments were repeated three times, and the average values were taken.

### Plate colony formation

Approximately 1500 U2OS cells or 143B cells per well were seeded into six-well plates with 1.5 ml of complete medium. Fresh medium was gently replaced approximately every 4 days. After 12 days, the cells were fixed with 4% paraformaldehyde (Servicebio, Wuhan, China) and stained with 0.1% crystal violet (Solarbio, Beijing, China) for 20 minutes. Clonal clusters containing more than 50 cells were manually counted. The experiments were repeated three times.

### Wound healing assay

143B or U2OS cells were seeded into a six-well plate with 3 horizontal lines marked with useful markers on the back and cultured in a cell incubator for 24 hours to spread the cells throughout the whole six-well plate. A sterile 200 µl pipette tip was then used to make 2 scratches perpendicular to the marking line. The medium was gently replaced with fresh medium, and the cells were cultured for another 24 hours (39). The widths of the wounds in the different treatment groups were observed and photographed under a microscope (Nikon Ti2-U, Japan) at 0 and 24 hours after scratching. The wound healing rate was calculated as the percentage of gap closure: (0 hours−24 hours)/0 hours × 100%. For the assays related to FAC, the medium in each well was replaced with medium with or without 100 µM FAC after the scratch was completed. The other experimental steps were the same as before, and all the experiments were repeated three times.

### Transwell invasion and migration assays

NEST culture inserts (Wuxi NEST Biotechnology Co., Ltd.) with 8.0 µm pore size polycarbonate membrane 24-well plates were used in Transwell invasion and Transwell migration assays. For the invasion assay, 30 µg of Matrigel matrix (Corning, USA) was added to each upper chamber. Two hundred microliters of serum-free 143B or U2OS (5 × 10^4^) cell suspension was seeded into each upper chamber, and 600 µL of complete medium containing 10% FBS was added to the lower chamber and cultured in an incubator for 24 hours. The cells were fixed with 4% paraformaldehyde (Servicebio, Wuhan, China) and stained with 0.1% crystal violet (Solarbio, Beijing, China). The cells that did not cross the chamber membrane were removed, and three fields of view were randomly selected under a microscope at 100× magnification to take pictures and count the number of cells. For the assays related to FAC, 600 µL of complete medium with or without 100 µM FAC was added to the lower chamber, and the remaining steps were the same as before. For the Transwell migration assay, the cell seeding concentration was 2 × 10^4^ per well, no Matrigel matrix was added to the upper chamber, and the remaining experimental steps were the same as those for the Transwell invasion assay. All the experiments were repeated independently three times (39).

### Total iron quantification

A total iron colorimetric assay kit (E-BC-K880-M, Elabscience, Wuhan, China) was used to assess total intracellular iron. Approximately 1 × 10^6^ cells were collected, lysed on ice, and centrifuged at 15000×g for 10 minutes, after which the supernatant was collected. The supernatant and chromogenic solution were mixed, added to a 96-well plate and incubated at 37°C for 40 minutes. The OD value of each well was measured at 593 nm via a microplate reader (SpectraMax Plus384, Molecular Devices, USA), and the total iron content of each well was calculated via a standard fitting curve.

### *In vivo* xenograft experiments

The animal experiment was approved by the Ethics Committee on Animal Experiments of Shandong University Qilu Hospital (permit number DWLL-2023-011). Five-week-old (17–20 g) male BALB/c nude mice were purchased from Zhejiang Vital River Laboratory Animal Technology Co., Ltd. (Zhejiang, China) and maintained in a specific pathogen-free (SPF) environment with free access to food and water. One week later, the mice were randomly divided into a Lenti-shCtrl group and a Lenti-sh*TFRC* group. A 100 µl 1:1 mixture of PBS and Matrigel matrix (Corning, USA) containing approximately 5 ×10^6^ *TFRC*-knockdown or nonknockdown 143B cells was subcutaneously injected into the right flank of each nude mouse. Tumor size and mouse body weight were measured every 5 days after injection. All the mice were euthanized 3 weeks later, and the tumor tissues were removed, weighed, snap-frozen in liquid nitrogen and stored at -80°C. Harvested samples were subjected to Western blotting and qRT–PCR analysis. The volume of the tumor was calculated via the following formula: V=length × width^2^/2.

### Statistical analysis

All the data are expressed as the mean ± standard error of the mean (SEM) unless otherwise indicated. All *in vitro* experiments were independently repeated at least 3 times under the same conditions. Statistical significance was assessed via the unpaired two-tailed Student’s t test or repeated measurement analysis of variance or nonparametric test, and P<0.05 was considered statistically significant. SPSS 22.0 was used for statistical analysis of all the data, and GraphPad Prism 9 was used for graphical presentation of the data.

## Results

### *TFRC* is overexpressed in osteosarcoma and is associated with poor overall survival

**Figure 1:**
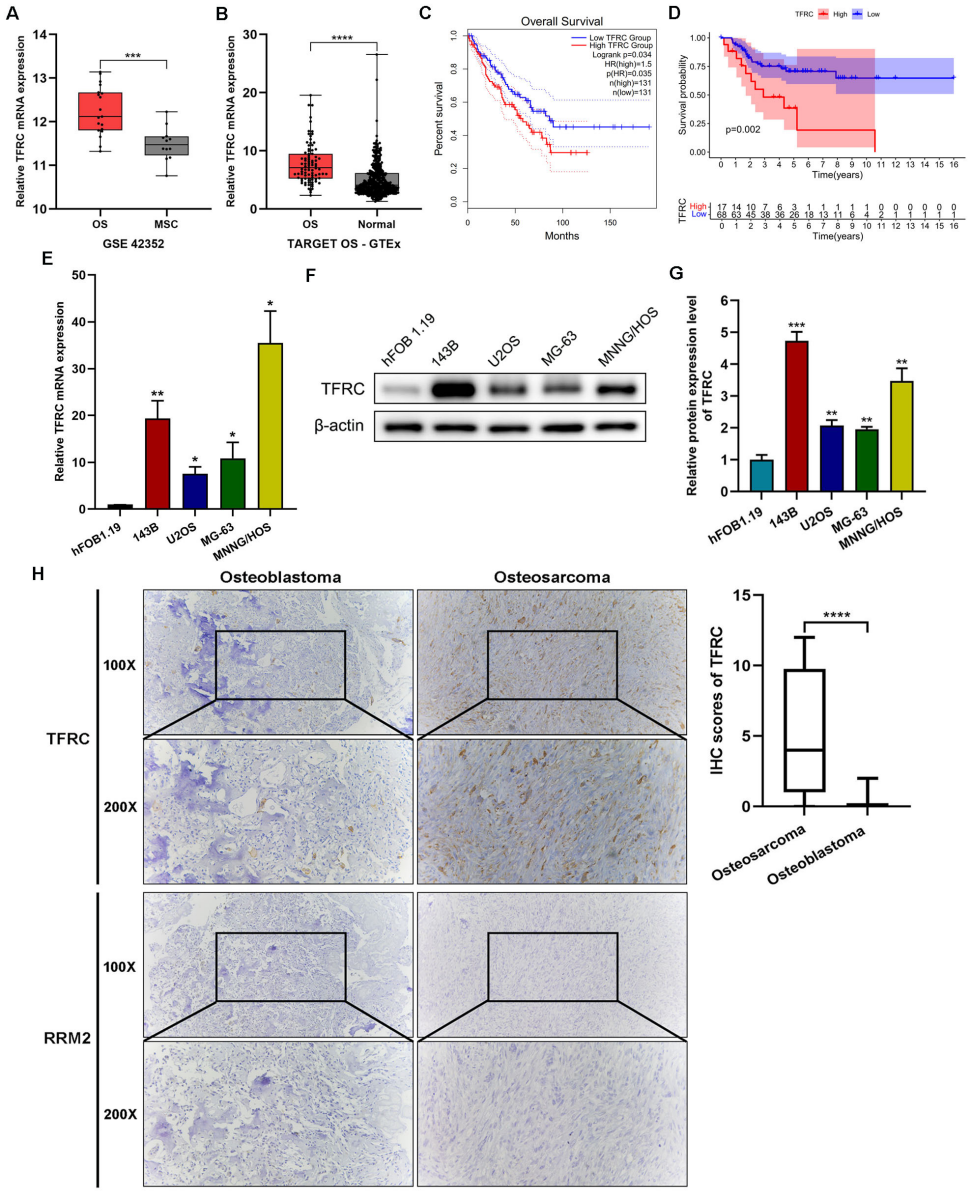
*TFRC* is overexpressed in osteosarcoma and is associated with poor overall survival. **(A)** The expression of *TFRC* in osteosarcoma cell lines (OS, n=19) was significantly greater than that in mesenchymal stem cells (MSCs, n=12) in the GSE42352 dataset. **(B)** The expression of *TFRC* in the integrated datasets of TARGET-OS (n=88) and GTEx (n=396). **(C)** Sarcoma patients (n=262) with high *TFRC* gene expression had significantly shorter overall survival than those with low *TFRC* gene expression according to the GEPIA online data analysis platform. **(D)** The TARGET-OS dataset (n=88) indicated that the overall survival of OS patients with low *TFRC* expression was better than that of those with high *TFRC* expression. **(E-G)** Compared with the human osteoblast cell line hFOB1.19, the human osteosarcoma cell lines 143B, U2OS, MG-63 and MNNG/HOS presented greater expression of *TFRC* at the protein and mRNA levels, as determined by quantitative real−time PCR (qRT–PCR) and Western blotting analysis (n=3). **(H)** Immunohistochemical (IHC) staining of human osteosarcoma tissues (n=30) and human osteoblastoma tissues (n=12) revealed that the staining intensity and degree of *TFRC* were significantly greater in osteosarcoma tissues than in osteoblastoma tissues. There was a significant difference in the IHC staining scores of *TFRC* between the two groups. However, *RRM2*-specific staining was notably weak in both osteosarcoma and osteoblastoma tissues. All the data are presented as the means ± SEMs. *P< 0.05, **P< 0.01, ***P< 0.001, ****P< 0.0001.

Several studies have suggested that *TFRC* is abundantly expressed in liver, breast, lung and colon cancer cells and that this increased expression may be associated with poor prognosis in different types of cancer (13, 15). First, we analyzed the data from several publicly available databases. The results revealed that in the GSE42352 dataset, the expression of *TFRC* in osteosarcoma cell lines (n=19) was significantly greater than that in mesenchymal stem cells (n=12) (P<0.001; **Figure 1A**). In the integrated datasets of TARGET-OS (n=88) and GTEx (n=396), *TFRC* was also significantly overexpressed in osteosarcoma tissues compared with normal skeletal muscle tissues (P<0.0001; **Figure 1B**). In addition, by using the GEPIA online data analysis platform, we found that in the sarcoma dataset (SARC, n=262), patients with high *TFRC* gene expression had significantly shorter overall survival than did those with low *TFRC* gene expression (P=0.035; **Figure 1C**). The overall survival status of the 88 patients in the TARGET-OS dataset was analyzed by R software, and the results indicated that the overall survival of patients with low *TFRC* expression was better (P=0.002; **Figure 1D**).

To confirm the expression and function of *TFRC* in osteosarcoma cell lines and tissues, qRT–PCR and Western blotting were performed on 4 kinds of human osteosarcoma cell lines (143B, U2OS, MG-63 and MNNG/HOS) and the human osteoblast cell line hFOB1.19. The results revealed significantly greater expression of *TFRC* mRNA and protein in osteosarcoma cell lines than in the human osteoblast cell line hFOB1.19 (**Figures 1E-G**). In addition, we used IHC staining to assess the protein expression of *TFRC* and *RRM2* in human osteosarcoma tissues (n=30) and osteoblastoma tissues (n=12) as controls. Compared with that in control tissues, the expression of *TFRC* in osteosarcoma tissues was significantly greater, and the IHC staining score significantly differed (P<0.0001; **Figure 1H**). These data indicate that *TFRC* is overexpressed at the mRNA and protein levels in osteosarcoma cells. However, to our surprise, IHC experiments showed that *RRM2*-specific staining was notably weak in both osteosarcoma and osteoblastoma tissues (**Figure 1H**).

### *TFRC* knockdown inhibits the proliferation, migration, and invasion of human OS cells

**Figure 2:**
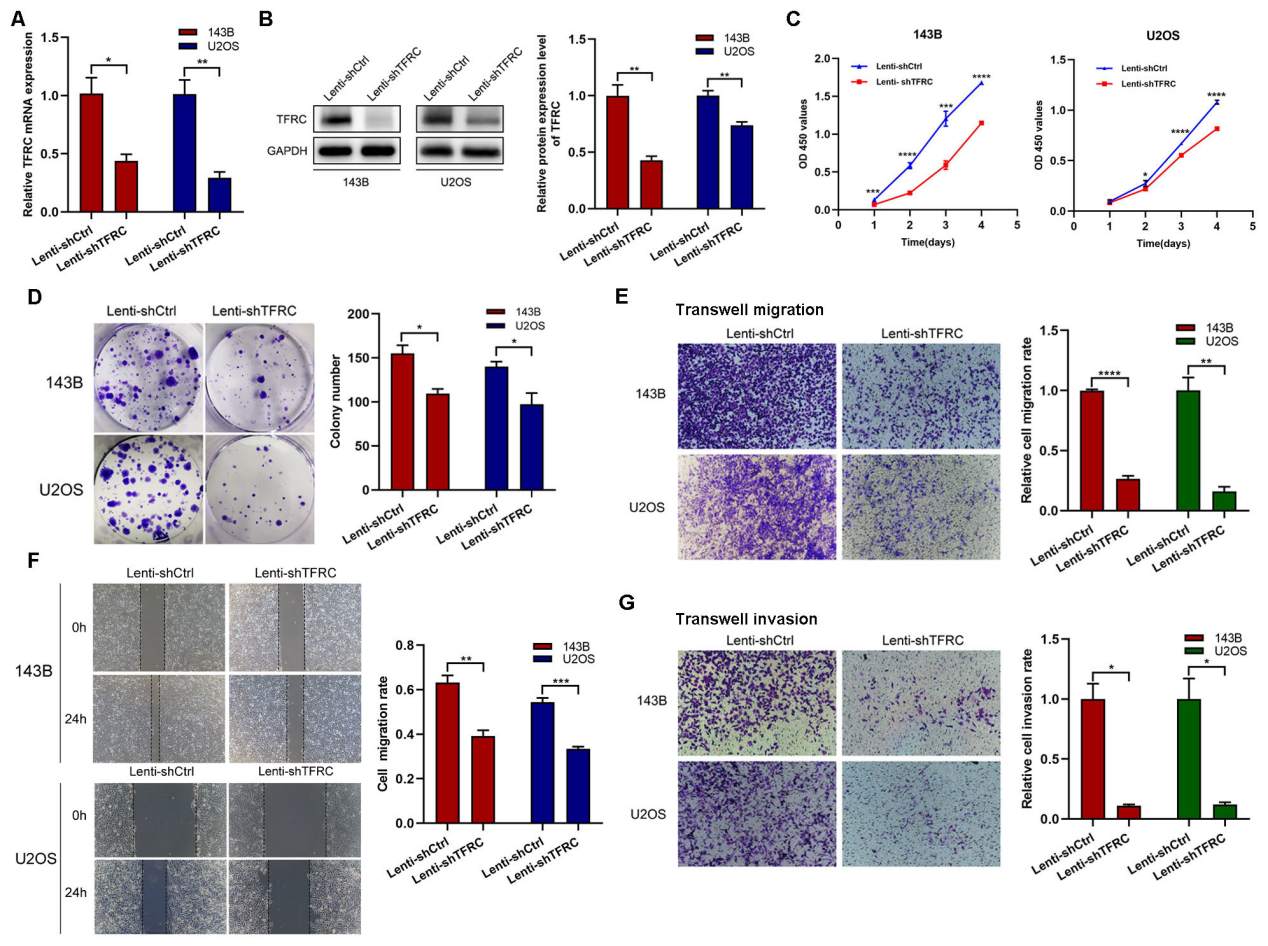
*TFRC* knockdown inhibits the proliferation, migration, and invasion of human OS cells. **(A, B)** qRT–PCR and Western blotting confirmed that the mRNA and protein levels of *TFRC* were successfully decreased in the 143B and U2OS cell lines by lentivirus-mediated *TFRC* small hairpin RNA (shRNA). **(C, D)** Cell Counting Kit-8 (CCK-8) and plate colony formation assays revealed that, compared with that in control cells, *TFRC* knockdown significantly suppressed cell proliferation (n=3). **(E, F)** Wound healing and Transwell migration assays were used to assess the migration ability of the cells, and the results revealed that, compared with the control conditions, the knockdown of *TFRC* significantly decreased the wound healing rate and number of migrated cells (n=3). **(G)** Knockdown of *TFRC* significantly inhibited the invasion ability of 143B and U2OS cells, as determined by a Transwell invasion assay (n=3). Except for the CCK-8 assay data, which are presented as the means ± SDs, all other experimental data are presented as the means ± SEMs. *P< 0.05, **P< 0.01, ***P< 0.001, ****P< 0.0001.

In previous experiments, we reported that *TFRC* was abnormally overexpressed in human osteosarcoma cells. Therefore, to investigate the role of *TFRC* in OS, we selected the 143B and U2OS cell lines, which have relatively high *TFRC* expression, for further experiments. We transfected 143B and U2OS cells with shRNAs and screened stable *TFRC*-knockdown cell lines. qRT–PCR and Western blotting analysis revealed that the mRNA and protein levels of *TFRC* were successfully decreased in these two OS cell lines (**Figures 2A, B**). We subsequently conducted CCK-8 and colony formation assays to investigate the effect of *TFRC* knockdown on OS cell proliferation. As shown in **Figure 2C**, the absorbance of *TFRC*-knockdown cells was significantly lower than that of control cells. Moreover, the number of colonies formed in the *TFRC*-knockdown groups was significantly lower than that in the control groups (P<0.05; **Figure 2D**). These findings suggest that the knockdown of *TFRC* attenuates the proliferation of OS cells. Wound healing and Transwell migration assays were used to assess the migration ability of the cells. The results revealed that, after *TFRC* knockdown, migration was significantly weakened compared with that of control cells (all P < 0.01; **Figures 2E, F**). In addition, a Transwell invasion assay with Matrigel was used to assess the invasive ability of human OS cells. The number of invasive cells in the experimental group was also significantly lower than that in the control group (**Figure 2G**). Taken together, these findings indicate that *TFRC* knockdown inhibits the proliferation, migration and invasion ability of human OS cells.

### *RRM2* is positively correlated with *TFRC* and is related to the poor prognosis of sarcoma patients

**Figure 3:**
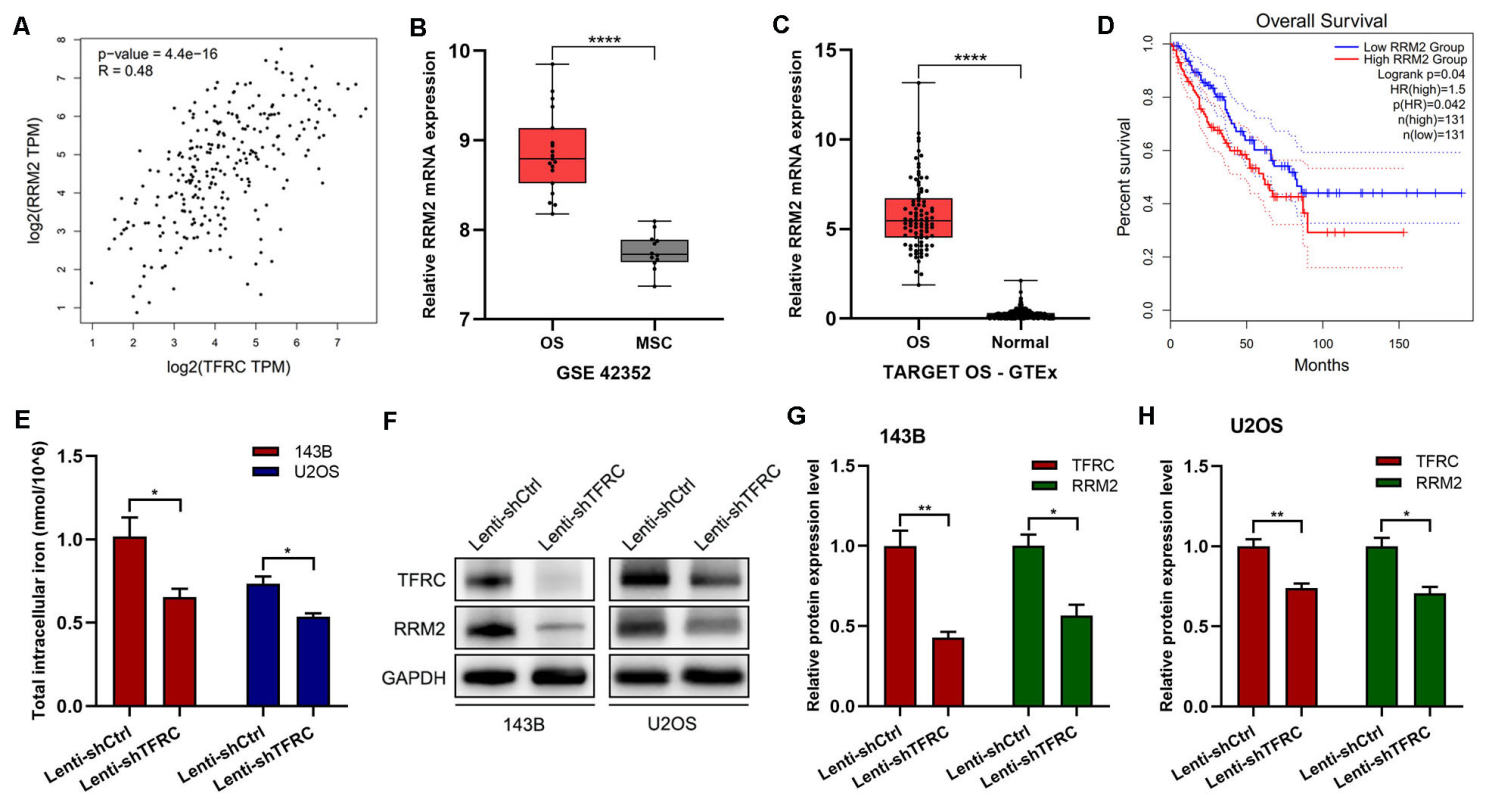
*RRM2* and *TFRC* are significantly positively correlated. **(A)** The GEPIA platform revealed a positive correlation between *TFRC* and *RRM2*. **(B-C)** The GSE42352 (n=19) and TARGET OS-GTEx (n=88) datasets revealed that the expression of *RRM2* in osteosarcoma cells was significantly greater than that in control cells. **(D)** The GEPIA online data analysis platform revealed that in the sarcoma dataset (SARC, n=262), high *RRM2* gene expression was associated with poor overall survival. **(E)** A total iron colorimetric assay kit was used to assess total intracellular iron, and the OD value was measured at 593 nm via a microplate reader. Knockdown of *TFRC* significantly reduced the total intracellular iron content in 143B and U2OS cells. **(F-H)** Western blotting results also revealed that with the knockdown of *TFRC*, *RRM2* was significantly downregulated at the protein level. All the data are presented as the means ± SEMs, *P< 0.05, **P< 0.01, ***P< 0.001, ****P< 0.0001.

TFR1 is a crucial carrier for iron transport into cells (21), and iron is an indispensable metal element for the synthesis of activated *RRM2*. As a subunit of RNR, *RRM2* plays important roles in DNA synthesis, cell proliferation and cancer development (28). Therefore, we used the GEPIA platform to explore the correlation between *TFRC* and *RRM2*, and the results, shown in **Figure 3A**, indicated a significant positive correlation between *RRM2* and *TFRC*. We then analyzed the expression of *RRM2* and its relationship with survival through public databases. The results showed that in the GSE42352 dataset, the expression of *RRM2* in osteosarcoma cell lines (n=19) was significantly greater than that in mesenchymal stem cells (n=12) (P<0.0001; **Figure 3B**). In the integrated datasets of TARGET-OS (n=88) and GTEx (n=396), *RRM2* was also significantly overexpressed in osteosarcoma tissues compared with normal skeletal muscle tissues (P<0.0001; **Figure 3C**). In addition, by using the GEPIA online data analysis platform, we found that in the sarcoma dataset (SARC, n=262), patients with high *RRM2* gene expression had significantly shorter overall survival than did those with low *RRM2* gene expression (P=0.042; **Figure 3D**).

### Knockdown of *TFRC* can lead to a decrease in the total intracellular iron content and the downregulation of *RRM2* expression

To further investigate the mechanism of the decreased proliferation, migration and invasion of human OS cells caused by the knockdown of *TFRC*, we assessed the total intracellular iron content and *RRM2* expression in OS cells. The results revealed that the total intracellular iron concentration in 143B cells after *TFRC* knockdown was 0.654 ± 0.051 nmol/10^6^ and that in control cells was 1.016 ± 0.115 nmol/10^6^, which was a significant difference (P=0.045). Similar results were obtained in the U2OS cell line: 0.537 ± 0.020 nmol/10^6^ for Lenti-sh*TFRC* cells vs. 0.736 ± 0.042 nmol/10^6^ for Lenti-shCtrl cells (P=0.013). These results indicated that the knockdown of *TFRC* significantly reduced the total iron content in the cytoplasm of human OS cells (**Figure 3E**). In addition, the Western blotting results revealed that with the knockdown of TFRC, *RRM2* was significantly downregulated at the protein level (**Figures 3F–H**).

### Upregulation of *RRM2* rescues the decrease in the proliferation, migration, and invasion of human OS cells after *TFRC* knockdown

**Figure 4:**
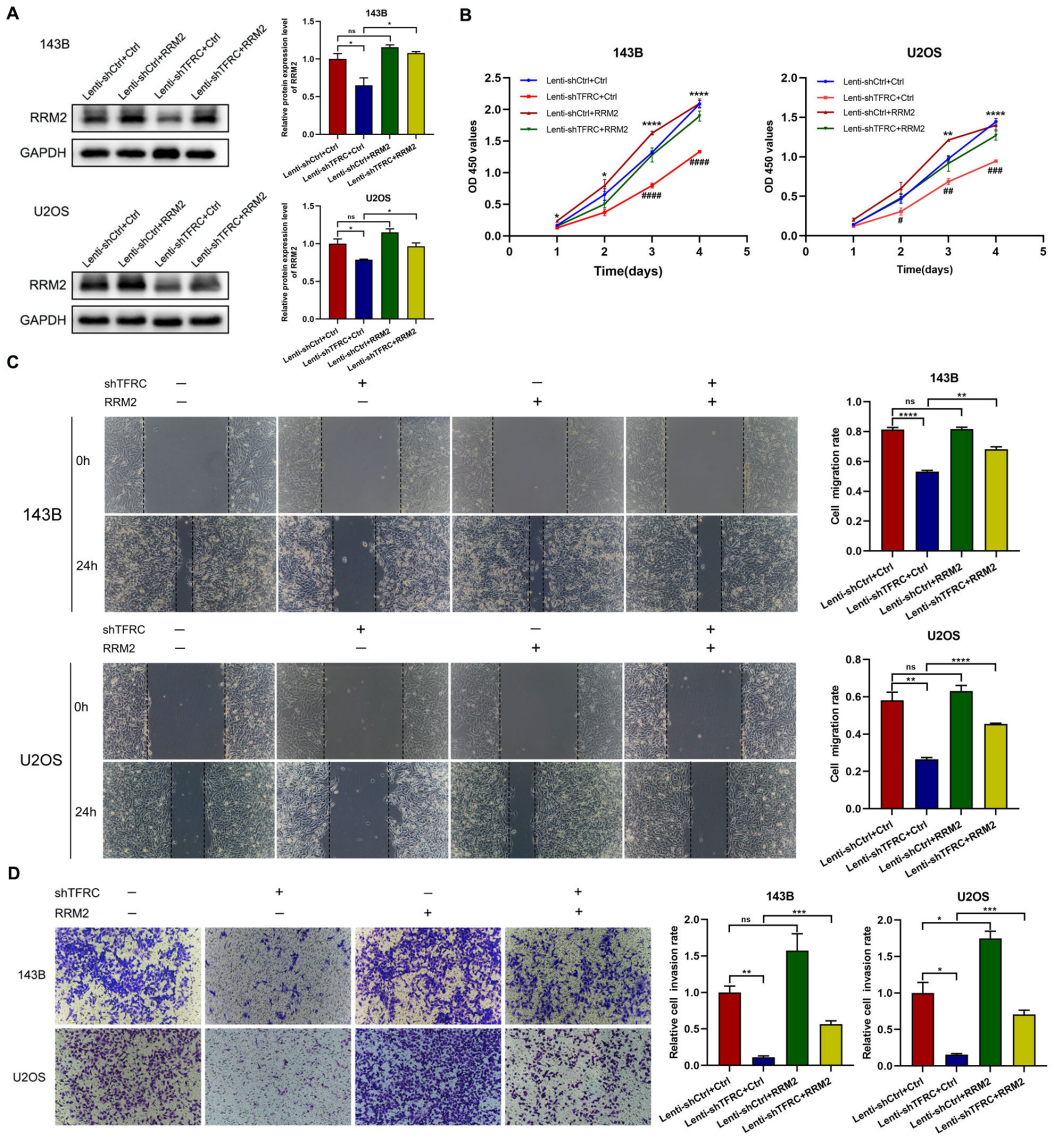
The upregulation of *RRM2* rescues the decreases in the proliferation, migration, and invasion of human OS cells after *TFRC* knockdown. **(A)** Cells in the Lenti-sh*TFRC* group (*TFRC* knockdown) and Lenti-shCtrl group (no *TFRC* knockdown) were transfected with the *RRM2* overexpression plasmid or control plasmid. The Western blotting results revealed that *RRM2* was upregulated by the *RRM2* overexpression plasmid. **(B)** CCK-8 assays revealed that *RRM2* upregulation significantly increased the proliferation ability of OS cells with *TFRC* knockdown compared with that of nonupregulated cells, especially on days 3 and 4. The asterisks indicate comparisons between the Lenti-shCtrl+Ctrl plasmid group and the Lenti-sh*TFRC*+Ctrl plasmid group, and the pound signs indicate comparisons between the Lenti-sh*TFRC*+Ctrl plasmid group and the Lenti-sh*TFRC*+*RRM2* plasmid group. **(C)** Wound healing assays revealed that by overexpressing *RRM2*, cell mobility after *TFRC* knockdown was also significantly improved. **(D)** The *RRM2*-overexpressing group had more invasive OS cells than did the control group after *TFRC* knockdown. Except for the CCK-8 assay data, which are presented as the means ± SDs, all other experimental data are presented as the means ± SEMs. *P< 0.05, **P< 0.01, ***P< 0.001, ****P< 0.0001, #P< 0.05, ##P< 0.01, ###P< 0.001, ####P< 0.0001.

To demonstrate the role of *RRM2* in human OS cells, we constructed an *RRM2* overexpression plasmid and a control plasmid and successfully transfected the plasmid into OS cells. The Western blotting results revealed that *RRM2* protein expression was increased after transfection with the *RRM2* overexpression plasmid, especially in *TFRC*-knockdown OS cells (**Figure 4A**). We subsequently performed a series of related experiments. The results of the CCK-8 assay revealed that, in OS cells with *TFRC* knockdown, the absorbance of the *RRM2*-overexpressing group was greater than that of the *RRM2*-untreated group, especially on days 3 and 4 (**Figure 4B**). A wound healing assay revealed that when *RRM2* was overexpressed, cell mobility after *TFRC* knockdown was also significantly improved (all P < 0.01; **Figure 4C**). In addition, we used a Matrigel Transwell invasion assay to assess changes in invasion ability. As shown in **Figure 4D**, the *RRM2*-overexpressing group had more invasive OS cells than did the control group after *TFRC* knockdown. In summary, these findings further confirm that the upregulation of *RRM2* rescues the decrease in the proliferation, migration and invasion of OS cells after *TFRC* knockdown.

### FAC upregulates *RRM2* expression and promotes the proliferation, migration, and invasion of OS cells after *TFRC* knockdown

**Figure 5:**
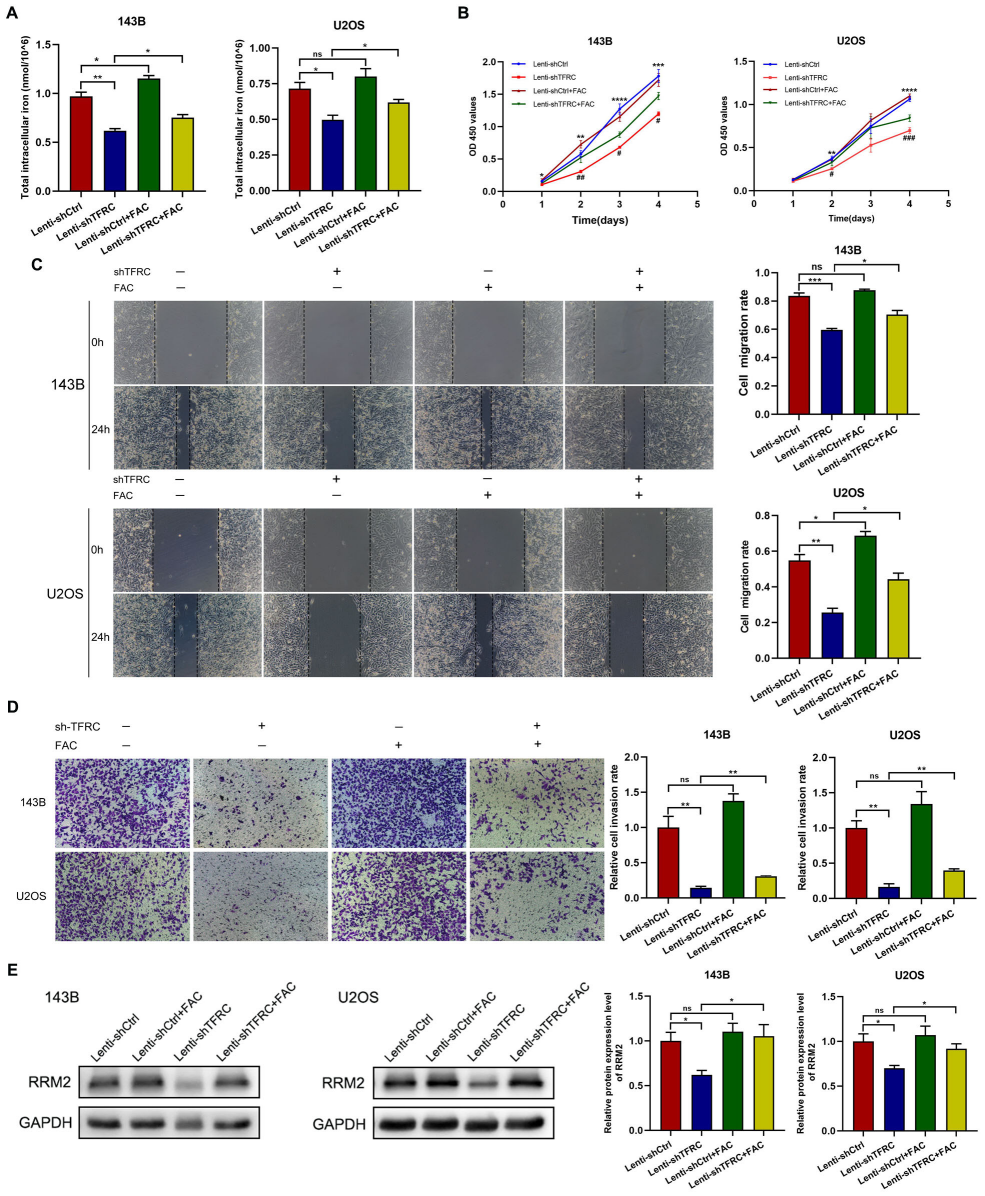
Ferric ammonium citrate (FAC) upregulates *RRM2* expression in OS cells after *TFRC* knockdown and promotes proliferation, migration, and invasion. **(A)** Cells were incubated with complete medium supplemented with or without 100 μM FAC, and the total intracellular iron concentration was increased 48 hours after the addition of FAC compared with that in the absence of FAC. **(B)** CCK-8 assays revealed that, compared with the absence of FAC, the addition of FAC increased the proliferation of OS cells with *TFRC* knockdown. The asterisks indicate comparisons between the Lenti-shCtrl group and the Lenti-sh*TFRC* group, and the pound signs indicate comparisons between the Lenti-sh*TFRC* group and the Lenti-sh*TFRC*+FAC group. **(C)** Wound healing assays revealed that the addition of FAC increased the mobility of OS cells after *TFRC* knockdown. **(D)** The group treated with FAC had more invasive OS cells than did the control group after *TFRC* knockdown. **(E)** The addition of FAC increased the protein expression level of *RRM2*, as determined by Western blotting, after *TFRC* knockdown. Except for the CCK-8 assay data, which are presented as the means ± SDs, all other experimental data are presented as the means ± SEMs. *P< 0.05, **P< 0.01, ***P< 0.001, ****P< 0.0001, #P< 0.05, ## P< 0.01, ### P< 0.001.

To explore the interactions among *TFRC*, iron and *RRM2* and their effects on OS cell function in depth, we further investigated the role of iron in human OS cells on the basis of our existing experiments. Because knockdown of *TFRC* reduced the total iron content in OS cells, we treated the cells with complete medium with or without 100 μM FAC. Compared with that in OS cells without FAC, total intracellular iron in OS cells was increased 48 hours after the addition of FAC (**Figure 5A**). The results of the CCK-8 assay revealed that, in OS cells with *TFRC* knockdown, the absorbance of cells treated with FAC was significantly greater than that of cells without FAC (**Figure 5B**). A wound healing assay revealed that the migration of cells after *TFRC* knockdown was also significantly improved by supplementation with exogenous iron (**Figure 5C**). Moreover, the Transwell invasion assay results revealed that the addition of FAC increased the invasion ability of OS cells after *TFRC* knockdown (**Figure 5D**). To our surprise, Western blotting revealed that the addition of FAC increased the protein expression level of *RRM2* after *TFRC* knockdown (**Figure 5E**). These findings suggest that supplementation with exogenous iron may increase the expression of *RRM2*, leading to increased proliferation, migration, and invasion of *TFRC*-knockdown OS cells.

### Knockdown of *TFRC* suppresses tumor formation in nude mice

**Figure 6:**
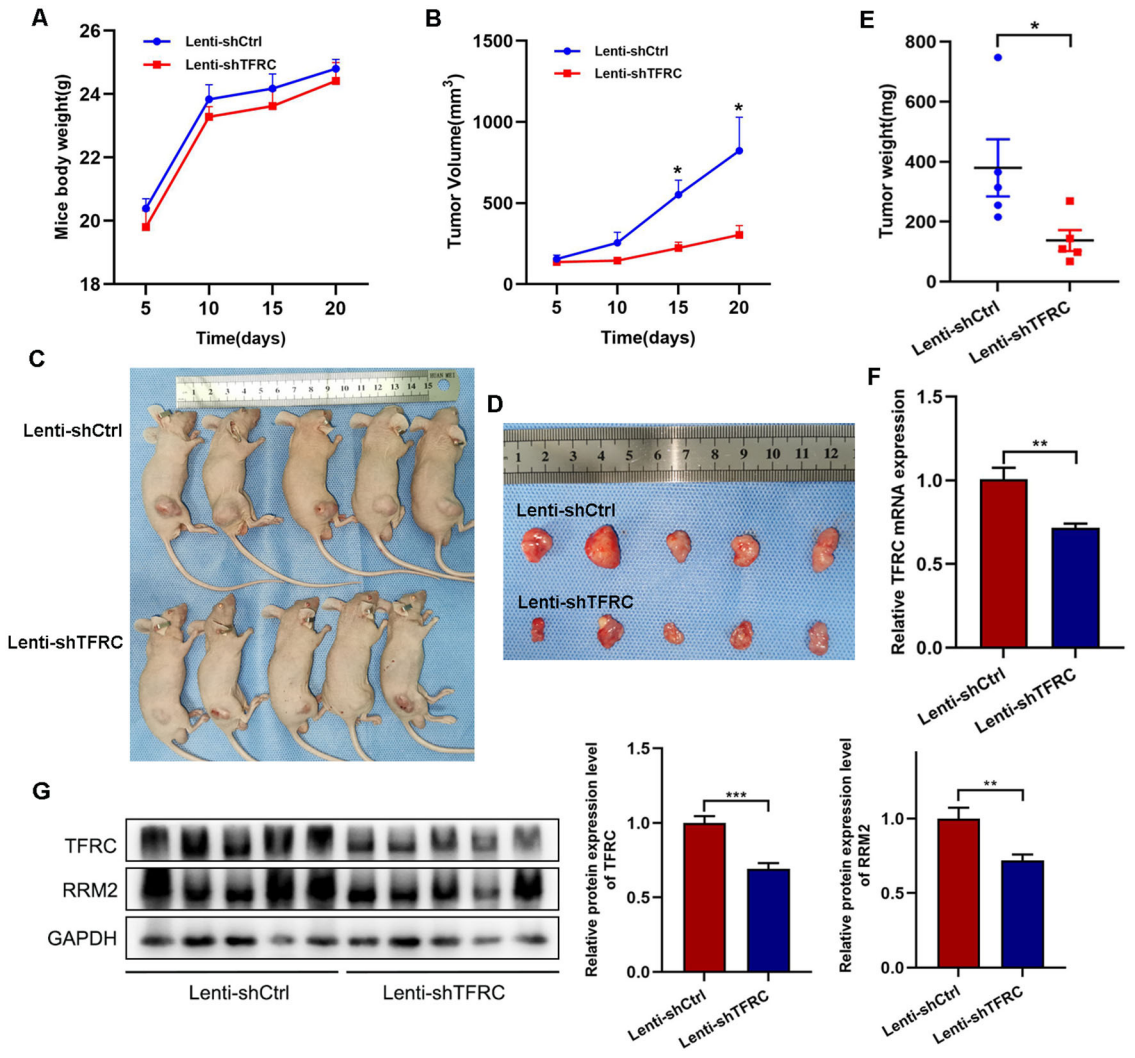
Xenograft tumor formation of 143B cells with *TFRC* knockdown in nude mice. **(A)** There was no significant difference in body weight among the groups of mice (n=5). **(B, E)** Compared with the control treatment, *TFRC* knockdown reduced the tumor volume and weight (n=5). **(C, D)** Each nude mouse was subcutaneously injected with 5 × 10^6^ *TFRC*-knockdown or nonknockdown 143B cells (n=5 per group). The mice were euthanized 3 weeks later, and the tumors were removed and photographed. (**F, G)** qRT–PCR and Western blotting were performed on the removed tumors (n=5). All the data are presented as the means ± SEMs, *P< 0.05, **P< 0.01, ***P< 0.001.

To assess whether the proliferation of human OS cells *in vivo* was affected by *TFRC* knockdown, stable *TFRC*-knockdown or nonknockdown 143B cells (5 × 10^6^/mouse) were injected subcutaneously into nude mice, and all the mice were euthanized 3 weeks later. The results revealed that all of the mice had an increase in body weight, but there was no significant difference between the two groups (**Figure 6A**). Through measurement and calculation, the tumor volume in the control group was significantly greater than that in the *TFRC*-knockdown group, especially after day 15 (**Figures 6B–D**). The weights of the tumors in the control group (379.70 ± 95.44 mg) were also notably greater than those in the *TFRC*-knockdown group (137.62 ± 35.03 mg) (**Figure 6E**). Finally, qRT–PCR and Western blotting were performed on the removed tumors, and the results revealed that *TFRC* was still downregulated at the protein and mRNA levels in the *TFRC*-knockdown group (**Figures 6F, G**). Meanwhile, Western blotting assay revealed that *RRM2* was also downregulated at the protein level (**Figure 6G**). These results suggest that the knockdown of *TFRC* inhibits the tumorigenicity of human OS cells *in vivo*.

## Discussion

Despite some progress in surgical interventions and neoadjuvant chemotherapy in recent years, the prognosis of osteosarcoma is still unsatisfactory. For patients diagnosed with metastasis, mainly lung metastasis (40), the 5-year survival rate sharply decreases to 20% (7) because of early metastasis of osteosarcoma and chemotherapy resistance (6). Therefore, exploring new molecular mechanisms and new therapeutic targets is still the focus of osteosarcoma research. Through various experiments, we found that *TFRC* is generally highly expressed in osteosarcoma cells and affects the proliferation, migration and invasion of osteosarcoma cells by regulating the total intracellular iron level and *RRM2* expression.

*TFRC* has been confirmed to be highly expressed in a variety of malignant tumors and provides a sufficient iron source to support the rapid proliferation of tumor cells by promoting iron endocytosis, which is also considered an important promoting factor for tumor cell growth and poor prognosis (16, 41, 42). Several previous studies have successfully inhibited the proliferation and invasion of various tumor cells, such as glioblastoma, lymphoma, ovarian cancer, and hepatocellular carcinoma cells, by inhibiting the *TFRC* gene (43–46). In this study, we analyzed several public databases and reported that *TFRC* is overexpressed in osteosarcoma and that this overexpression is associated with poor overall survival in osteosarcoma patients. Our subsequent experiments confirmed that *TFRC* is overexpressed at the mRNA and protein levels in four osteosarcoma cell lines: MNNG/HOS, U2OS, MG-63, and 143B. Compared with 12 samples of osteoblastoma tissues, 30 samples of clinical osteosarcoma tissues also presented high expression level of *TFRC* as determined by immunohistochemical staining. Moreover, after *TFRC* knockdown via lentivirus-mediated shRNA, the proliferation, migration and invasion ability of OS cells were significantly reduced, and *TFRC* knockdown effectively inhibited the tumorigenicity of OS cells in *in vivo* xenograft experiments. Similar results have also been reported by other scientists. Feng et al. knocked down *TFRC* in nasopharyngeal carcinoma, which inhibited the proliferation, migration, invasion and epithelial–mesenchymal transition of nasopharyngeal carcinoma cells (47). These findings provide an experimental basis for *TFRC* as a potential therapeutic target for OS. Surprisingly, in addition to the above conclusions, we observed an unexpected phenomenon during the research process. We unexpectedly detected the significant upregulation of *TFRC* in osteoclasts from osteoblastoma tissues. As an important cell type in bone, osteoclasts directly participate in calcium metabolism and bone remodeling, and the dysfunction of osteoclasts may be closely related to osteoporosis. Whether *TFRC* regulates the function of osteoclasts through certain mechanisms, affecting bone metabolism balance and leading to the development of osteoporosis, rheumatoid arthritis, Paget’s disease of bone, etc., is also a topic worthy of further in-depth research.

The mammalian RNR contains two subunits, α and β, which are encoded by *RRM1* and *RRM2* (or *P53R2*) genes respectively. The α subunit contains a catalytic site (C), an activity site (A) and a specificity site (S) for substrate selection. Within each β subunit resides both a μ-oxo-bridged di-nuclear iron cluster (Fe-O-Fe) and a tyrosyl radical (Y-O•), which constitutes the core structure essential for RNR catalytic activity (27, 28, 48, 49). However, due to its long half-life, *RRM1* protein levels remain constant throughout the cell cycle, whereas *RRM2* levels fluctuate (27, 28, 50, 51). *RRM2* is an important enzyme involved in DNA synthesis and repair and plays crucial roles in cell proliferation, DNA replication, and maintenance of normal growth (52). *RRM2* has been confirmed to be overexpressed in hepatocellular carcinoma, breast cancer, Ewing sarcoma and atypical teratoid rhabdoid tumors which is related to tumor progression and poor prognosis, and can also be used as a key marker for aggressive tumors (53–56). Inhibiting the expression or activity of *RRM2*, or both, can interfere with the dNTP pool, hinder DNA repair and replication, and thus increase the anticancer activity of chemotherapeutics, especially DNA-damaging drugs (52). Souglakos et al. reported that *RRM2* mRNA expression in lung adenocarcinoma patients was positively correlated with the response to gemcitabine combination therapy, and compared with patients with high *RRM2* mRNA expression, those with low *RRM2* expression had greater drug sensitivity (57). Some scientists have also reported that the application of the antitumor drug didox, a derivative of hydroxyurea, can target *RRM2* and inhibit its activity by quenching tyrosine free radicals at the enzyme active site, thereby suppressing the proliferation of liver cancer cells. However, the addition of ferric ammonium citrate reduced the inhibitory activity of didox in a dose-dependent manner (32). Our analysis of public databases revealed that *RRM2* is overexpressed in osteosarcoma and that this overexpression is associated with poor overall survival in osteosarcoma patients. Western blotting revealed that *RRM2* protein expression was also significantly reduced after *TFRC* was knocked down in OS cells. The transfection of *RRM2*-overexpressing plasmids partially reversed the decrease in the proliferation, migration, and invasion ability of osteosarcoma cells caused by *TFRC* knockdown. These findings suggest that there is a certain regulatory relationship between *TFRC* and *RRM2*, which may be one of the important pathways regulating the biological functions of OS cells. However, to our surprise, IHC experiments showed that *RRM2*-specific staining was notably weak in both osteosarcoma and osteoblastoma tissues. We evaluated several potential explanations for this finding. First, differences in cellular structure and physiological state across tissues can significantly impact epitope accessibility. In some tissue types, target epitopes may become masked or undergo post-translational modifications, preventing effective antibody binding and consequently reducing immunohistochemical detection sensitivity (58, 59). Our study included 30 OS specimens without pathological subtype classification and potential differences in *RRM2* antigen status across various OS subtypes may account for this result. Secondly, the antibody we used may have exhibited weak antigen-binding affinity in IHC assays, resulting in poor tissue staining and making it unsuitable for immunohistochemical applications. Additionally, we cannot rule out potential technical influences from tissue processing, fixation, or antigen retrieval procedures. However, we recommend exercising caution when selecting IHC methodologies for future *RRM2* studies in osteosarcoma.

As an essential metal cofactor for *RRM2*, iron plays a key role in the metabolism, proliferation and growth of tumor cells (16). Iron deficiency reduces the bioavailability of iron-related cofactors, thereby reducing the catalytic activity of many iron-dependent enzymes, including RNR (60). In their study on Parkinson’s disease, Key et al. reported that iron deprivation reduced *RRM2* mRNA expression in mouse and human fibroblasts, leading to decreased availability of dNTPs for mtDNA repair and increased release of superoxide from uncoupled mitochondria, resulting in mtDNA damage (61). Our research revealed that *TFRC* knockdown leads to a decrease in the total intracellular iron content, while the addition of FAC increases not only the total intracellular iron content in OS cells after *TFRC* knockdown but also the expression of *RRM2*, effectively rescuing the decrease in OS cell proliferation, migration, and invasion caused by *TFRC* knockdown. Although the physiological correlation of *RRM2* downregulation with low-iron conditions is not yet clear, Puig S et al. suggested that this is a cellular strategy to reduce the level of apo-*RRM2* dimers (60).

However, this study also has some limitations. The utilization and regulation of iron in eukaryotic cells involves a complex network of proteins. While our study focused on *TFRC*, it did not account for other critical components of iron regulation, including ferritin, transferrin, ferroportin, iron regulatory proteins (IRPs), iron-responsive elements (IREs), and divalent metal transporter 1 (DMT1), etc., all of which play essential roles in cellular iron metabolism (9, 62, 63). In addition, there are biological and genetic differences between cell line models and patient populations. Although we used widely recognized osteosarcoma cell lines such as 143B and U2OS, these cells may lose tumor heterogeneity characteristics in long-term passaging (64). The growth microenvironment of xenografted OS cells in nude mice differs from the tumor microenvironment in humans, which also plays a crucial role in tumor progression, invasion, metastasis, and angiogenesis (65, 66). Consequently, the generalizability and translational applicability of the findings from the cell line models and xenograft models to patient populations are worth considered. Moreover, our clinical sample size was limited, and we did not classify the pathological subtypes of osteosarcoma, which may have affected the accuracy of the IHC results.

## Conclusion

**Figure 7:**
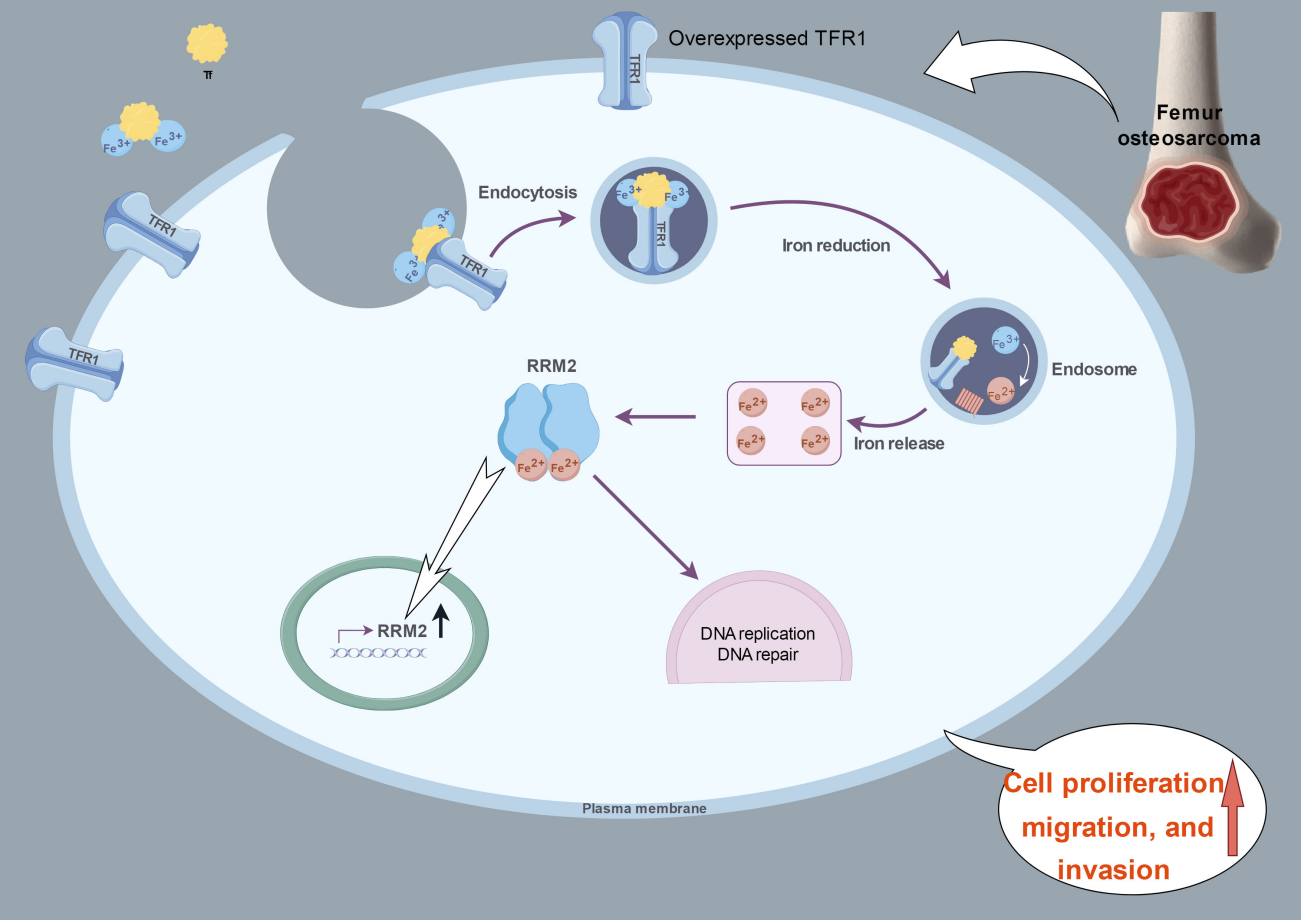
A proposed schematic model: in OS cells, *TFRC* is overexpressed and increases the intracellular iron content and *RRM2* expression. This image summarizing this mechanism was created at www.figdraw.com.

Taken together, our experimental results suggested that OS cells regulate proliferation, migration, and invasion by overexpressing *TFRC*, which increases the transport of iron into the cell and increases the expression and activity of *RRM2* (**Figure 7**). In this study, we not only verified the overexpression of *TFRC* in osteosarcoma and its important role in iron metabolism but also discovered that *TFRC* can participate in the proliferation, migration, and invasion activities of osteosarcoma by regulating *RRM2*, which provides potential molecular targets and new therapeutic strategies for the treatment of osteosarcoma. However, *TFRC* may also regulate the biological functions of OS cells through many other complex pathways. How does *TFRC* affect DNA replication and repair in the absence of iron? Are there any other iron-dependent enzymes that act simultaneously? What specific role does iron play in different DNA metabolism steps? All these aspects need to be further studied.

## Data Availability

The datasets presented in this article are readily available, and requests to access the datasets should be directed to meddocrgq@163.com.
